# The Bidirectional Relationship Between Picky Eating and Eating Dinner Alone in Japanese Adolescents: A Longitudinal Study Using RI-CLPM

**DOI:** 10.3390/nu17172882

**Published:** 2025-09-06

**Authors:** Miao Wu, Akira Ishida

**Affiliations:** Graduate School of Agricultural Science, Kobe University, Kobe 657-8501, Japan; wumiao19960823@gmail.com

**Keywords:** picky eating, eating dinner alone, adolescence, RI-CLPM, family meals, longitudinal study

## Abstract

**Background**: Picky eating often persists from childhood into adolescence, yet its temporal relation to solitary dinners is unknown. We examined the bidirectional links between eating dinner alone and picky eating across three developmental stages in a nationwide Japanese cohort. **Methods**: A total of 1389 two-parent families from the Japanese Longitudinal Study of Children and Parents participated in the study (grades 4–6 in 2015; grades 7–9 in 2018; grades 10–12 in 2021). Eating dinner alone (four-point scale) was analyzed as a two-part variable (binary ever/never + continuous frequency); picky eating was ordinal (four categories). A Bayesian Random Intercept Cross-Lagged Panel Model (RI-CLPM) with a two-part specification for eating alone was used to assess cross-lagged, autoregressive, and covariate paths; covariates were gender, grade sequence, parental education, and household income. **Results**: A single cross-lagged path proved significant: adolescents who ate dinner alone at least once per week in junior high school showed higher-than-their-own-average picky eating in high school, and the reverse paths were non-significant. Picky eating and the binary indicator of eating alone exhibited moderate positive autoregression, whereas the continuous frequency of solitary dinners showed a negative carry-over from Wave 1 to Wave 4, consistent with regression-to-the-mean. Boys, students in higher grades, and adolescents from higher-income households were more prone to solitary dinners, whereas girls exhibited higher trait-like levels of picky eating; parental education showed no significant associations. **Conclusions**: Frequent solitary dinners in junior high school may set the stage for later elevations in picky eating, underscoring the preventive value of shared family meals before early adolescence.

## 1. Introduction

Picky eating (sometimes called fussy eating) is a common childhood behavior characterized by a limited variety of accepted foods and refusal of many familiar and unfamiliar items [1]. Longitudinal data suggest that roughly 39% of children are identified as picky eaters at some point in early childhood [2]. A survey in Japan found that about half of junior high school students reported having foods they did not like [3], and recent polls showed that more than 60% of parents were concerned about their children’s picky eating behavior [4].

Picky eating has been demonstrated to be harmful to health. Research has shown correlations between picky eating and being underweight or having poor growth [5]. At age 13, picky eaters consume 23% less vegetables and 14% less fruit than non-picky peers [6]. Children labeled as picky by their parents are reported to show higher levels of negative emotions [7]. As eating habits formed early in childhood and adolescence are likely to persist into adulthood [5,8], understanding the determinants of picky eating is essential for designing effective interventions.

In this regard, solitary eating has received increasing attention, as eating alone has become increasingly common in many societies. For example, research in the United States has shown that about 20% of adolescents frequently have meals alone [9]. In Japan, some studies have warned of a worrying decline in the frequency of family dinners [10,11]. Only 12.6% of children have breakfast together with all family members, representing a decline of about 10% compared to findings reported 20 years ago [11]. Research among Japanese school children reveals that while more than 80% of elementary school students eat breakfast (81%) and dinner (87%) with family members, the proportion falls gradually from junior (70% and 81%) to senior high school (55% and 81%) [12]. A review has indicated that frequent family meals are related to improved eating habits and reduced overweight in children [13].

Several cross-sectional studies have reported an association between eating alone and picky eating. Adjusted odds of U.S. adolescents who often ate alone were significantly higher for overweight or obese adolescents, compared with normal- or underweight adolescents (OR = 1·6), and for additional daily intake frequency of junk food/sugary drinks (OR = 1·1) [9]. The Japanese study found that there was a significant difference in terms of children’s picky eating between the group with high frequency of eating with a guardian and the group with low frequency, with values of 71.0% and 53.6%, respectively [14]. Adolescents who used to eat breakfast alone consumed fewer vegetables, fruit, and fish than those who ate with parents or guardians (trend *p*-value < 0.01) [15]; compared to people who ate alone more than 2 days per week, those who ate alone less often were more likely to consume a balanced meal (62.3% > 42.4%) [16]. A study conducted in China also found that the frequency of eating alone among adolescents was significantly associated with the intake of processed foods (B = 0.72) and sugary beverages (B = 0.22) [17]. However, these cross-sectional studies precluded any inference about the causality between eating alone and picky eating.

From a causality perspective, there may be a bidirectional effect between eating alone and picky eating. On the one hand, eating alone may decrease exposure to role modeling and supervision by parents or peers, leading to narrower food acceptance and increased picky eating behavior [18,19,20]. In the absence of such modeling and supervision, children are more likely to rely on snacks or fast food and form picky eating habits [21,22,23]. It has also been noted that habitual solitary eating can lead to children choosing only favorite foods, which, over time, may develop into a “fixed diet” rather than a balanced diet [24]; on the other hand, picky eating itself may prompt children to avoid family meals. Some parents reported preparing separate meals or allowing their children to skip a shared dinner when they rejected the family menu [25,26]. In addition, variables such as the child’s gender [11,19,27,28,29], school grade [19], parental educational level [30,31], and household income [32] may also influence eating alone and picky eating behaviors. In summary, it is difficult to accurately assess the direct causal relationship between eating alone and picky eating without considering bidirectional causality and controlling for the interference of other variables.

However, to the best of our knowledge, only one longitudinal study has examined the prospective association between the frequency of family dinners and picky eating. Berge et al. (2023) found that a higher baseline frequency of family dinners was associated with improved diet quality (B = 0.33, *p* < 0.01) and reduced picky eating (B = −0.10, *p* < 0.001) at follow-up 18 months later [33]. However, the study did not test whether picky eating predicted later changes in the frequency of family meals.

Although there is currently no specific longitudinal research examining the bidirectional causal relationship, we draw on findings from studies investigating the bidirectional influences between other factors and adolescent eating behaviors, such as loneliness and disordered eating behaviors [34], or self-esteem and problematic eating behaviors [35,36]. We argue that the bidirectional effects of eating dinner alone and picky eating behavior are at the within-person level, and that the traditional Cross-Lagged Panel Model (CLPM) cannot distinguish between within-person changes over time and between-person differences [37]. Therefore, the Random Intercept Cross-Lagged Panel Model (RI-CLPM) is applied, which effectively separates the sources of within-person and between-person variance [38,39].

The present study makes a novel contribution by directly testing bidirectional relations between eating dinner alone and picky eating across important developmental transitions in Japanese youth. Unlike prior work that relied on cross-sectional designs or examined only one causal direction, we used three waves of panel data spanning late elementary (grades 4–6), junior high (grades 7–9), and high school (grades 10–12) and applied an RI-CLPM adapted for ordinal measures. This approach separates within-person changes from stable between-person differences. We also include key covariates (child sex, school-grade sequence, parental education, and household income) to reduce confounding factors. The specific aims of the study are as follows:

- Aim 1: Estimate the within-person prospective effect of increases in eating dinner alone on subsequent changes in picky eating.
- Aim 2: Estimate the within-person prospective effect of increases in picky eating on subsequent changes in eating dinner alone (the reverse direction).
- Aim 3: Separate within-person from between-person variance to determine whether the observed bidirectional relationship accurately reflects the dynamics within the individual.
- Aim 4: Add the key time-invariant sociodemographic factors (sex, grade sequence, parental education, household income) to assess their impact on between-person differences.

## 2. Materials and Methods

This study employed data from the Japanese Longitudinal Study of Children and Parents in the 2015 (Wave 1), 2018 (Wave 4), and 2021 (Wave 7) waves, which were conducted by the Benesse Educational Research and Development Institute in collaboration with Institute of Social Science, University of Tokyo. Since its introduction in 2015, the survey has been administered annually between July and September to students in grades 1 to 12 (elementary through high school) and their parents, who were recruited from across Japan. In Wave 1, samples were drawn from each grade level, and data were collected through mailed questionnaires. Thereafter, the survey continued to track the established sample (excluding students who had graduated from high school) each year, while introducing a new cohort of first-grade students and their families. According to the report [40], sample attrition and bias related to demographic or socioeconomic characteristics are relatively low. Although the survey is conducted every year, this analysis focuses on the 2015, 2018, and 2021 waves, which are the only years that include data on children’s eating behaviors such as picky eating and eating dinner alone.

The analytic sample was restricted to two-parent households in which the child was between grades 4 and 6 at the time of the 2015 wave. Children in grades 1 to 3 were excluded because, for these grades, only parents completed the questionnaire, not children, and therefore the data would be less comparable. Similarly, children in grade 6 or above in 2015 were excluded because they would likely have already graduated from high school by 2021, resulting in missing data in later waves. Single-parent households were also excluded as they had far greater odds of children having dinner alone, which could lead to confounding of the relationship of interest. To facilitate longitudinal analysis, the data from the three time points (2015, 2018, and 2021) were converted to person-year format. In total, 4300 two-parent households with children in grades 4 to 6 in 2015 responded to all three waves. After excluding cases with missing responses for key variables, 1389 cases remained for analysis.

A four-point scale was used to assess the frequency at which children eat dinner alone on weekdays: “4–5 days a week”, “2–3 days a week”, “1 day a week”, and “Never”. This variable was treated both as binary (e.g., “Never” vs. all other categories) and as an ordinal variable in different parts of the analysis (details provided in the Statistical Analyses section). Picky eating behavior was assessed through children’s self-reports of frequency, rated on a four-point scale: “Very often”, “Sometimes”, “Rarely”, and “Never”. This measure was treated as an ordinal variable.

To account for individual background characteristics, several covariates were controlled in the analysis. Parental years of education and household income were used to measure socioeconomic background. Household income was employed as a continuous variable to capture the economic component, calculated as the average of the midpoint values of parental reports of income categories for the past year across the 2015, 2018, and 2021 waves. Parental education was also treated as a continuous variable and coded by the highest educational level that the parents had: 9 for junior high school, 12 for high school, 14 for vocational school/junior college/technical college, 16 for university, and 18 for graduate school. The child’s gender and sequence of school grades were included as additional covariates. Descriptive statistics for all the variables are presented in Table 1.

### Statistical Analyses

All data management and descriptive statistics were conducted with R (version 4.4.2). The RI-CLPM with Mplus (version 8.11) was used to examine the directional relationship between eating dinner alone and picky eating. Given that the distribution of eating dinner alone was semi-continuous, RI-CLPM was specified as a two-part model and estimated using Bayesian methods. The estimation of the model, evaluation of fit index, and assessment of the significance of parameter estimates followed the procedure outlined by Muthén et al. (2024) [41].

Muthén et al. (2024) proposed a two-part modeling approach for ordinal indicators with strong floor effects [41]. This approach divides the variable into two components: a binary part indicating whether the response is in the bottom category (e.g., 0 = never eats dinner alone; 1 = at least sometimes eats alone), and an ordinal part for values above the floor, coded as missing if the binary component is zero. These two components are allowed to have distinct associations with other variables in the model. The Bayesian estimator was used for the simultaneous modeling of both components under this framework.

In the current study, eating dinner alone was assessed in Mplus as a two-part variable: (1) a binary indicator of whether the child ever had dinner alone weekly (0 = never; 1 = at least once per week), and (2) a continuous factor representing frequency, with zero points regarded as missing and all non-zero values retained. These two parts were estimated jointly using Bayesian estimation, and the model estimated featured random intercepts, within-time correlations, autoregressive paths across waves, and cross-lagged paths between eating dinner alone and picky eating.

Model fit was evaluated using the posterior predictive *p*-value (PPP), as outlined by Muthén et al. (2024), with ≤0.05 indicating poor fit and proximity to 0.5 indicating good model–data fit [41]. The statistical significance of model estimates was assessed by 95% credibility intervals, derived from posterior standard deviations; those whose intervals did not include zero were interpreted as significant. Lastly, covariates were included in the best-fitting model to investigate how they relate to the random intercept factors, reflecting stable differences between persons.

Ethical review and approval were waived for this study because we used publicly available secondary data from the Japan Social Science Data Archive, Center for Social Research and Data Archives, Institute of Social Science, University of Tokyo.

## 3. Results

The RI-CLPM converged after 30,000 iterations and demonstrated a good model fit (PPP = 0.502), indicating that the model effectively explained inter-individual stability and intra-individual temporal dynamics.

As displayed in Table 2, only one cross-lagged effect reached statistical credibility: adolescents who ever ate dinner alone in junior high (Wave 4) were more likely to exhibit picky eating frequencies above their own long-term average in high school (Wave 7; Est. = 0.165, 95% CI [0.000, 0.327]). Because the lower bound is exactly zero, this finding should be interpreted as borderline significant and weighed against the modest effect size.

Autoregressive coefficients for picky eating were positive and credible across both intervals (W1 → W4: Est. = 0.166; W4 → W7: Est. = 0.295), indicating that deviations from an individual’s own mean level tended to persist over time. Similarly, the binary component of eating dinner alone exhibited considerable rank-order stability (W1 →W4: Est. = 0.203; W4 → W7: Est. = 0.356). In contrast, the continuous component showed a negative carry-over from Wave 1 to Wave 4 (Est. = −0.513), suggestive of regression-to-the-mean effects during the primary-to-junior-high transition; this pattern did not extend into the later interval.

After simultaneously entering gender (reference = boys), school grade, parental education, and household income as covariates, we regressed all three random intercepts on this full set of predictors in a single step. The model converged after 110,600 iterations, and exhibited acceptable fit (PPP = 0.396). Results are shown in Table 3.

Eating Dinner Alone—Binary: Girls were significantly less likely than boys to eat dinner alone (Est. = −0.219). Higher grade level (Est. = 0.180) and higher household income (Est. = 0.031) were also associated with a greater likelihood of eating dinner alone.

Eating Dinner Alone—Continuous: Among adolescents who had ever eaten dinner alone, grade level was positively related to the frequency of doing so (Est. = 0.089); no other covariates reached significance.

Picky Eating intercept: Girls exhibited a higher trait-like mean level of picky eating behavior than boys (Est = 0.221). Grade level, parental education, and household income showed no significant associations.

## 4. Discussion

This study is the first to apply an RI-CLPM to test whether eating dinner alone and picky eating mutually influence one another during adolescence. After controlling for stable between-person differences, we observed one significant cross-lagged effect: adolescents who ate dinner alone at least once per week in junior high school were more likely to exceed their own long-term average level of picky eating behavior in high school. In contrast, the reverse pathway—from picky eating early on to eating dinner alone later—was not observed. Beyond this cross-lagged path, both picky eating and the binary indicator of eating dinner alone (ever vs. never) displayed moderate autoregressive stability across waves, whereas the continuous frequency of eating dinner alone exhibited a negative carry-over from late elementary to junior high school. Regarding covariates, girls had higher trait-like levels of picky eating than boys. In contrast, boys, higher grade level, and greater household income were associated with a higher likelihood of ever eating dinner alone. Among adolescents who had ever eaten dinner alone, a higher grade level was also associated with more frequent solitary dinners. Parental education was unrelated to any outcome.

This study is the first, to our knowledge, to apply an RI-CLPM and demonstrate that adolescents who frequently eat dinner alone in junior high school are more likely to exceed their own baseline level of picky eating behavior in high school. This finding aligns with extensive evidence on the benefits of shared family meals. Longitudinal studies indicate that regular family dinners are associated with higher subsequent intake of fruits and vegetables and lower odds of picky eating [18,33]. Collectively, these studies suggest that the absence of parental or peer modeling and supervision during meals may lead adolescents to rely on “safe” (i.e., familiar, non-novel) convenience foods, thereby limiting dietary variety and reinforcing picky eating tendencies [18,19,20].

Contrary to our bidirectional hypothesis, picky eating did not significantly predict subsequent avoidance of family dinners; in other words, the paths from earlier picky eating to later solitary dining were not significant. This may be because, in Japan, shared family meals occupy a central place in the national shokuiku (food education) agenda. The 2005 Basic Law on Shokuiku explicitly set a target of increasing family-meal frequency, and the government has monitored this indicator annually to evaluate policy effectiveness [10]. The expected evaluation of targets in 2015 is 10 times or more/week [10]. Within this cultural and policy context, adolescents who are picky eaters are still likely to take part in family meals. Consequently, instances of eating dinner alone in our sample are more plausibly attributable to scheduling conflicts or personal preference rather than to picky eating itself [42]. This background helps explain why we found no evidence for a “picky eating → eating dinner alone” pathway.

Picky eating displayed moderate, positive autoregressive effects across both intervals, corroborating the view that food fussiness is a relatively stable trait once established [27]. A recent twin study likewise documented high stability of food neophobia and narrow food preferences from early childhood through adolescence [43]. Among the covariates, gender was the only demographic characteristic that showed a significant association with the random intercept of picky eating, and, in the cross-lagged paths, only the binary indicator of eating dinner alone during junior high school significantly predicted picky eating levels in high school. Overall, once picky eating habits are established, they appear relatively resistant to later contextual influences, underscoring the need to shift preventive efforts earlier—during the transition from late elementary to junior high school—and to promote regular family meals. Future intervention studies are needed to test this hypothesis directly.

The negative autoregressive coefficient of the continuous part of eating dinner alone from Wave 1 to Wave 4 signals a classic regression-to-the-mean pattern: during the transition to junior high school, adolescents’ frequency of eating dinner alone tends to drift back toward their own long-term average. A plausible reason is that, as students advance in grade, they both gain greater autonomy over daily choices and face new scheduling demands from cram lessons or club activities. Under these shifting circumstances, children whose solitary dinner frequency was above their own average in late elementary school may reduce the behavior as social opportunities expand, whereas those below their average may start eating alone more often because after-school commitments delay family meals. This explanation is still speculative; future longitudinal studies should track extracurricular time budgets and parent–child schedule mismatches to directly test the proposed mechanism.

Covariate effects aligned with prior research. Boys and adolescents from higher-income households were more likely to eat dinner alone. Gender differences in mealtime patterns have been documented elsewhere: for example, several studies report that boys eat dinner alone more often than girls [11,19,27]. Otsuka et al. (2020) suggested that boys purchase fast food after school or club activities, leaving fewer opportunities for family meals [19]. Higher household income also predicted a greater likelihood of eating dinner alone, consistent with adult data showing that wealthier families eat dinner together less frequently (adjusted odds ratio (ref: low-income households): middle = 0.76, high = 0.51; trend *p*-value < 0.001) [32]. This may reflect tighter work schedules or wider access to outside food options. By contrast, parental education showed no association with solitary dining, mirroring the finding that education level has little impact on the frequency of family dinners (adjusted odds ratio (ref: low-education households): middle = 0.89, high = 0.93; trend *p*-value = 0.531) [32].

Grade level showed a clear gradient: the likelihood and frequency of eating dinner alone rose steadily into grade 9 and grade 12, the critical “exam years.” Roughly 41% of junior high school and 27% of senior high school students attend private cram schools to prepare for entrance examinations [44], keeping them out late and prompting irregular, solitary dinners. Prior work likewise indicates that heavy academic and extracurricular loads foster a “late, irregular, and solitary” eating pattern among Japanese youth [44].

For picky eating, gender emerged as the sole significant predictor: girls exhibited higher levels of pickiness than boys. Household income and parental education were unrelated to the picky eating intercept. This pattern is partly consistent with the previous studies—several report no systematic gender gap in pickiness [8,45,46], while others suggest girls may report more diet-related concerns or lower perceived diet quality [19,28,29]. Regarding socioeconomic factors, the present study did not show income or education effects on picky eating. The review by del Campo et al. (2025) [31] found that some studies report no association of parental SES with pickiness [47], while others have shown that picky eaters are more often from families with low household income than non-picky eaters (42% vs. 31.8%, respectively; *Χ*^2^(1) = 9.97, *p* < 0.01) [30].

The present study also has some limitations. First, all eating behaviors were measured by self-report, which sometimes introduces shared method variance and response bias. Second, our sample consisted solely of Japanese two-parent families; cultural factors (e.g., norm of family meals in Japan, use of cram schools) might limit generalizability to other populations. Third, picky eating was measured based only on frequency, omitting other important dimensions such as specific food rejections, underlying motives, and severity. Because measurement choices can alter both prevalence estimates and theoretical interpretations, future studies should adopt multi-informant, multi-method designs.

To build upon the present findings and address the limitations of this study, several avenues for future research are recommended. First, the employment of multi-informant designs (e.g., incorporating parent reports) and objective measures of dietary intake would help to mitigate potential self-report biases and provide a more comprehensive assessment of picky eating behaviors. Second, expanding the measurement of picky eating beyond frequency to include dimensions such as specific food rejections and underlying motives could offer deeper insights into its developmental dynamics. Third, intervention studies are needed to directly test the causal implication that promoting shared family meals can prevent the exacerbation of picky eating, particularly during the critical transition to junior high school. Finally, cross-cultural comparative studies are essential to determine whether the pathways identified here, especially the null effect of picky eating on solitary dining, are specific to cultural contexts with strong family-meal norms (like Japan’s shokuiku (food education) agenda) or represent a more universal pattern.

## 5. Conclusions

This study highlights the importance of the mealtime environment in shaping adolescents’ eating patterns. Encouraging regular family (or supervised) meals during early adolescence may help prevent the emergence of picky eating habits in later teen years. At the same time, practitioners should recognize that picky eating appears relatively stable by adolescence, so prevention efforts should start early. These findings open avenues for public health strategies—such as promoting family dinners—aimed at improving the nutritional behaviors of youth.

## Figures and Tables

**Table 1:** Descriptive statistics.

Variable	Overall	Gender	
		Male	Female
	N = 1389 ^1^	N = 638 ^1^	N = 751 ^1^
Grade			
7th grade	497 (35.8%)	231 (36.2%)	266 (35.4%)
8th grade	426 (30.7%)	196 (30.7%)	230 (30.6%)
9th grade	466 (33.5%)	211 (33.1%)	255 (34.0%)
Mother_edu			
junior high school	5 (0.4%)	2 (0.3%)	3 (0.4%)
high school	294 (21.2%)	131 (20.5%)	163 (21.7%)
vocational school/junior college/technical college	678 (48.8%)	304 (47.6%)	374 (49.8%)
university	399 (28.7%)	193 (30.3%)	206 (27.4%)
graduate school	13 (0.9%)	8 (1.3%)	5 (0.7%)
Father_edu			
junior high school	24 (1.7%)	10 (1.6%)	14 (1.9%)
high school	377 (27.1%)	184 (28.8%)	193 (25.7%)
vocational school/junior college/technical college	228 (16.4%)	102 (16.0%)	126 (16.8%)
university	659 (47.4%)	297 (46.6%)	362 (48.2%)
graduate school	101 (7.3%)	45 (7.1%)	56 (7.5%)
Household_income	7.8 (3.4)	7.9 (3.5)	7.7 (3.3)
Picky_w1			
never	367 (26.4%)	190 (29.8%)	177 (23.6%)
rarely	438 (31.5%)	197 (30.9%)	241 (32.1%)
sometimes	381 (27.4%)	165 (25.9%)	216 (28.8%)
very often	203 (14.6%)	86 (13.5%)	117 (15.6%)
Picky_w4			
never	395 (28.4%)	212 (33.2%)	183 (24.4%)
rarely	462 (33.3%)	209 (32.8%)	253 (33.7%)
sometimes	352 (25.3%)	142 (22.3%)	210 (28.0%)
very often	180 (13.0%)	75 (11.8%)	105 (14.0%)
Picky_w7			
never	480 (34.6%)	228 (35.7%)	252 (33.6%)
rarely	440 (31.7%)	212 (33.2%)	228 (30.4%)
sometimes	313 (22.5%)	132 (20.7%)	181 (24.1%)
very often	156 (11.2%)	66 (10.3%)	90 (12.0%)
Eatalone_w1			
never	1283 (92.4%)	571 (89.5%)	712 (94.8%)
1 day/week	60 (4.3%)	38 (6.0%)	22 (2.9%)
2–3 days/week	36 (2.6%)	23 (3.6%)	13 (1.7%)
4–5 days/week	10 (0.7%)	6 (0.9%)	4 (0.5%)
Eatalone_w4			
never	1111 (80.0%)	494 (77.4%)	617 (82.2%)
1 day/week	125 (9.0%)	68 (10.7%)	57 (7.6%)
2–3 days/week	121 (8.7%)	57 (8.9%)	64 (8.5%)
4–5 days/week	32 (2.3%)	19 (3.0%)	13 (1.7%)
Eatalone_w7			
never	920 (66.2%)	408 (63.9%)	512 (68.2%)
1 day/week	154 (11.1%)	69 (10.8%)	85 (11.3%)
2–3 days/week	202 (14.5%)	107 (16.8%)	95 (12.6%)
4–5 days/week	113 (8.1%)	54 (8.5%)	59 (7.9%)

^1^ n (%); mean (SD).

**Table 2:** Estimates of two-part model paths for eating dinner alone and picky eating.

Model Path	Est.	Posterior S.D.	95% Credibility Interval	
			Lower	Upper
Rank-order stability paths				
Dinner Alone-B				
W1 → W4	0.203 *	0.104	0.001	0.410
W4 → W7	0.356 *	0.085	0.189	0.521
Dinner Alone-C				
W1 → W4	−0.513 *	0.238	−1.044	−0.068
W4 → W7	−0.261	0.178	−0.656	0.043
Picky				
W1 → W4	0.166 *	0.090	0.001	0.358
W4 → W7	0.295 *	0.069	0.155	0.425
B. Lagged paths				
Dinner Alone-B (W1) → Picky (W4)	0.020	0.094	−0.169	0.198
Dinner Alone-B (W4) → Picky (W7)	0.165 *	0.084	0.000	0.327
Dinner Alone-C (W1) → Picky (W4)	0.250	0.388	−0.553	1.010
Dinner Alone-C (W4) → Picky (W7)	−0.050	0.224	−0.511	0.381
Picky (W1) → Dinner Alone-B (W4)	0.106	0.106	−0.102	0.309
Picky (W4) → Dinner Alone-B (W7)	0.014	0.065	−0.111	0.146
Picky (W1) → Dinner Alone-C (W4)	−0.089	0.078	−0.242	0.060
Picky (W4) → Dinner Alone-C (W7)	0.036	0.062	−0.096	0.148

Note: Dinner Alone-B = Eating Dinner Alone (Binary); Dinner Alone-C = Eating Dinner Alone (Continuous); Picky = Picky Eating. Estimates marked with * indicate that the 95% credibility interval does not include zero.

**Table 3:** Demographic covariates predicting the random intercepts of Eating Dinner Alone (Binary and Continuous) and Picky Eating.

Covariate	Est.	Posterior S.D.	95% Credibility Interval	
			Lower	Upper
Random Intercept—Eating Dinner Alone (Binary)				
Gender	−0.219 *	0.061	−0.339	−0.100
Grade	0.180 *	0.035	0.109	0.248
Father_edu	−0.016	0.017	−0.049	0.017
Mother_edu	0.044	0.022	−0.001	0.087
Household_income	0.031 *	0.010	0.012	0.050
Random Intercept—Eating Dinner Alone (Continuous)				
Gender	−0.027	0.053	−0.132	0.077
Grade	0.089 *	0.031	0.027	0.150
Father_edu	−0.018	0.015	−0.047	0.011
Mother_edu	0.034	0.019	−0.003	0.072
Household_income	0.012	0.008	−0.005	0.028
Random Intercept—Picky Eating				
Gender	0.221 *	0.072	0.083	0.363
Grade	−0.037	0.038	−0.112	0.038
Father_edu	−0.035	0.020	−0.074	0.004
Mother_edu	−0.026	0.025	−0.075	0.022
Household_income	0.000	0.012	−0.024	0.023

Note: Estimates marked with * indicate that the 95% credibility interval does not include zero.

## Data Availability

Raw data are publicly available upon request from the Japan Social Science Data Archive, Center for Social Research and Data Archives, Institute of Social Science, University of Tokyo, Japan.
